# Complexed Linalool with Beta-Cyclodextrin Improve Antihypertensive Activity: Pharmacokinetic and Pharmacodynamic Insights

**DOI:** 10.3390/ph19010037

**Published:** 2025-12-23

**Authors:** Samuel Camargo, Carla Medeiros, Liliane Silva, Rafael Leonne Jesus, Fênix Araujo, Daniele Brito, Quiara Alves, Raiana Moraes, Valdeene Santos, Francine Azeredo, Adriano Araújo, Lucindo Quintans-Júnior, Darizy Silva

**Affiliations:** 1 Gonçalo Moniz Institute, Oswaldo Cruz Foundation (FIOCRUZ), Salvador 41745-715, Brazil; camargo.fisio2016@gmail.com (S.C.); fenixaaraujo@gmail.com (F.A.); danielesb20@gmail.com (D.B.); quiara.lovatti@gmail.com (Q.A.); rai.pharma@hotmail.com (R.M.); 2 Laboratory of Cardiovascular Physiology and Pharmacology, Federal University of Bahia, Salvador 40110-902, Brazil; barret.liliane@gmail.com (L.S.); rafaelleonne@gmail.com (R.L.J.); 3 Department of Pharmacology, Ribeirao Preto Medical School, Federal University of São Paulo, São Paulo 14049-900, Brazil; fiamaazevedo19@gmail.com; 4 Laboratory of Pharmacokinetic and Pharmacometrics, Federal University of Bahia, Salvador 40170-115, Brazil; enevieira@hotmail.com (V.S.); francinej@gmail.com (F.A.); 5 Center for Pharmacometrics and Systems Pharmacology, Department of Pharmaceutics, College of Pharmacy, University of Florida, Orlando, FL 32827, USA; 6 Graduate Program in Health Sciences, Federal University of Sergipe, Aracaju 49060-108, Brazil; adriasa2001@yahoo.com.br (A.A.); lucindojr@gmail.com (L.Q.-J.); 7 Department of Bioregulation, Federal University of Bahia, Salvador 40110-902, Brazil

**Keywords:** hypertension, linalool, antihypertensive efficacy, β-cyclodextrin

## Abstract

**Background:** Arterial hypertension (AH) remains a global health concern due to its multifactorial etiology, limited therapeutic success, and high cardiovascular risk. In this context, plant-derived compounds such as essential oils have gained attention as alternative strategies. The monoterpene (-)-linalool (LIN) demonstrates antihypertensive effects. However, its clinical application is hampered by poor solubility and low bioavailability. **Methods**: This study aimed to investigate the chronic cardiovascular effects of free LIN and its inclusion complex with β-cyclodextrin (LIN/β-CD) in spontaneously hypertensive rats (SHR) and normotensive Wistar rats. **Results**: Pharmacokinetic analysis showed that complexation with β-CD markedly improved LIN plasma exposure, increasing systemic bioavailability by approximately 20-fold and prolonging its circulation time. In acute assays, intravenous LIN and LIN/β-CD (50 mg/kg) reduced blood pressure in SHR, LIN induced bradycardia, and LIN/β-CD elicited a mild, non-significant tachycardia. Orally administered LIN/β-CD exerted superior antihypertensive effects compared to free LIN. In a 60-day chronic regimen, LIN/β-CD consistently maintained reduced arterial pressure, achieving levels comparable to normotensive controls, while free LIN produced transient effects. LIN/β-CD also significantly reduced the cardiac mass index in SHR, suggesting attenuation of hypertrophic remodeling. Vascular reactivity assays revealed enhanced endothelium-dependent and -independent relaxation and diminished vasoconstriction in LIN/β-CD-treated animals, indicating improved endothelial and smooth muscle function. Histological analyses confirmed the absence of cardiac or vascular injury in both treatment groups. **Conclusions**: In conclusion, the LIN/β-CD complex improves the pharmacokinetic profile and enhances the arterial morphology, antihypertensive and cardioprotective effects of linalool. These findings support its translational potential as a safe and effective oral formulation for the long-term management of hypertension and associated cardiovascular dysfunction.

## 1. Introduction

Arterial hypertension (AH) represents one of the leading global public health challenges, affecting over one billion individuals and contributing substantially to cardiovascular morbidity and mortality [1]. Despite therapeutic advancements, treatment adherence and adequate blood pressure (BP) control remain suboptimal, with only 21% of hypertensive patients achieving target BP levels [2].

Resistant hypertension (RH), defined as the failure to control BP despite the use of three or more antihypertensive agents, poses a significantly elevated risk of cardiovascular disease, target-organ damage, and increased mortality [3,4,5,6]. Moreover, the adverse effects associated with polypharmacy and the rising costs of treatment underscore the urgent need for innovative and effective therapeutic alternatives.

Spontaneously hypertensive rats (SHR) are a well-established model that closely reproduces the cardiovascular alterations observed in human hypertension, including progressive cardiac hypertrophy, endothelial dysfunction, and impaired vascular reactivity. These pathophysiological changes are largely driven by oxidative stress, dysregulated calcium handling, and reduced nitric oxide (NO) bioavailability, which together promote vascular remodeling and increased peripheral resistance [7,8,9,10,11]. NO, synthesized by endothelial nitric oxide synthase (eNOS), is essential for maintaining vascular tone and blood pressure homeostasis. Under hypertensive conditions, excessive oxidative stress leads to NO degradation and eNOS uncoupling, contributing to endothelial dysfunction and sustained vasoconstriction. Therefore, restoring NO-mediated signaling and reducing oxidative imbalance represent critical targets for therapeutic intervention in hypertension [12,13,14,15,16,17].

Given the limitations of current pharmacological approaches to AH and related cardiovascular disorders, there is an urgent need to explore alternative therapeutic strategies that are both effective and safe. In this context, natural products have emerged as promising candidates for the prevention and treatment of cardiovascular diseases. Their structural diversity and broad spectrum of biological activities offer a valuable platform for drug discovery. However, despite their therapeutic potential, further research is needed to elucidate the mechanisms of action, optimize formulations, and provide a robust scientific basis for their clinical use [18,19,20,21,22].

Among natural products, essential oils stand out due to their rich composition of bioactive molecules, primarily terpenes, extracted from aromatic plants [23]. Monoterpenes, the predominant constituents of many essential oils, exhibit a wide range of pharmacological effects, including anti-inflammatory [24,25], antinociceptive and analgesic [26], anxiolytic, antidepressant, and sedative properties [27,28], as well as vasorelaxant [29,30], and antihypertensive effect [31,32]. These multifunctional activities make them attractive candidates for the development of novel therapeutic agents targeting complex conditions such as hypertension and cardiovascular dysfunction.

**Figure 1:**
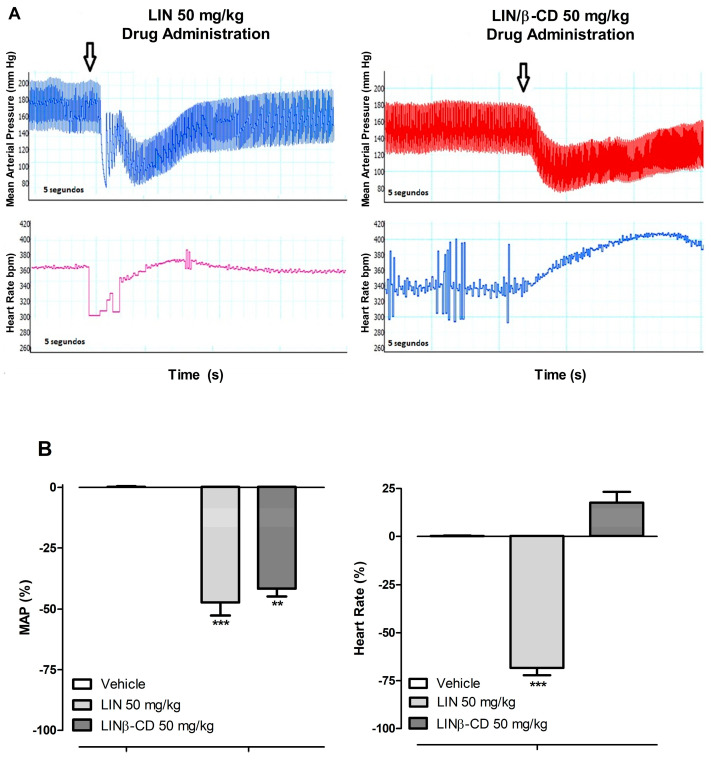
Hypotensive responses elicited by LIN and LIN/β-CD (50 mg/kg) following intravenous administration. (**A**) Representative original tracings of mean arterial pressure (MAP, mmHg) and heart rate (HR, bpm) recorded immediately after administration of LIN or LIN/β-CD. (**B**) Quantitative analysis of the hypotensive responses induced by vehicle, LIN or LIN/β-CD, expressed as percent changes in MAP (%MAP) and HR (%HR) relative to baseline values. Experimental groups consisted of vehicle (n = 5), LIN (n = 5), and LIN/β-CD (n = 5). Data are presented as mean ± S.E.M. ** *p* < 0.01, *** *p* < 0.001 vs. vehicle.

Linalool (LIN), chemically known as 3,7-dimethyl-1,6-octadien-3-ol (Figure 1), is a naturally occurring monoterpene alcohol characterized by a pleasant floral and citrus-like aroma. It is widely distributed in various medicinal and culinary plants, including bergamot (*Citrus bergamia*), jasmine (*Jasminum auriculatum*), basil (*Ocimum gratissimum*), and coriander (*Coriandrum sativum*) [19,20]. LIN has demonstrated multiple pharmacological effects, including significant antinociceptive and anti-inflammatory activity [33,34], as well as the ability to reduce allodynia in experimental models of neuropathic pain. Furthermore, its sedative and central nervous system effects have been associated with glutamatergic modulation, suggesting a potential role in the management of stress-related cardiovascular disorders [35].

Experimental studies have shown that LIN can relax contractions induced by prostaglandin F2α (PGF2α) in isolated rat aortic rings, as well as induce endothelium-dependent vasodilation in mice. These effects are believed to be mediated through the activation of soluble guanylate cyclase (sGC) and potassium channels, along with the inhibition of calcium influx [36]. Despite these promising pharmacological properties, the clinical application of LIN and other essential oil constituents is significantly limited due to their high volatility and pronounced lipophilicity (LogP ~ 3.3) [37], which negatively affect their bioavailability and therapeutic efficacy. These characteristics underscore the need to develop delivery systems that enhance LIN stability, bioactivity, and bioavailability, particularly in formulations pharmaceutics.

To overcome these limitations, drug delivery systems, particularly those based on cyclodextrins (CDs), have been increasingly employed to enhance the physicochemical and biological characteristics of natural compounds. Cyclodextrins are cyclic oligosaccharides composed of six to eight glucose units (α-, β-, and γ-CD, respectively) obtained through the enzymatic degradation of starch [38,39,40,41] (Figure 1). From a pharmaceutical standpoint, CDs are particularly valuable in oral drug delivery, as they can increase drug solubility, improve systemic absorption, and enable sustained or targeted release, while also reducing adverse effects [42].

Among the various types of cyclodextrins (CDs), β-cyclodextrin (β-CD), composed of seven glucose units, is the most widely used due to its ideal cavity size and cost-effectiveness compared to α- and γ-cyclodextrins [43] (Figure 1). β-CDs can form non-covalent inclusion complexes with guest molecules, stabilizing them through van der Waals forces, hydrophobic interactions, and hydrogen bonding [40]. These inclusion complexes enhance the solubility and stability of poorly water-soluble compounds, while also reducing their volatility and susceptibility to degradation, ultimately improving their pharmacokinetic and pharmacodynamic profiles [44]. Such improvements are particularly valuable for optimizing drug dosing and maintaining plasma concentrations with safe and effective therapeutic range, especially in the treatment of chronic conditions like AH.

The formation of inclusion complexes with β-CD has shown particular promise for monoterpenes such as LIN. Previous studies developed by our group suggest that this strategy not only improves the pharmacokinetic profile of LIN but also enhances its antihypertensive and pharmacological activities, as demonstrated in SHRs [32]. Furthermore, CDs are generally regarded as safe for oral administration for 21 days, exhibiting minimal toxicity. Considering the clinical need for chronic antihypertensive agents, our research group extended the treatment duration in both normotensive and hypertensive animal models to 60 days, with appropriate control groups, to rigorously evaluate the long-term therapeutic potential of these compounds—particularly pure linalool and its β-cyclodextrin complex—and to characterize the pharmacokinetic profile of this novel formulation. In view of the growing demand for innovative and safer therapeutic strategies in cardiovascular disease, the use of β-cyclodextrin to enhance linalool release emerges as a promising and rational approach.

The inclusion of LIN in β-CD complexes has the potential to overcome critical pharmacokinetic limitations commonly associated with natural compounds, such as low aqueous solubility, chemical instability, and reduced bioavailability. By improving these properties, such formulations may significantly broaden the therapeutic applicability of LIN as a natural antihypertensive agent. In this context, the present study aimed to evaluate the chronic cardiovascular effects of pure linalool and its β-cyclodextrin inclusion complex (LIN/β-CD), as well as to characterize their pharmacokinetic profiles, in spontaneously hypertensive rats (SHR), a well-established model of essential hypertension, and in normotensive Wistar rats.

## 2. Results

### 2.1. Pharmacokinetics Results

The pharmacokinetic parameters determined by non-compartmental analysis (NCA) following the administration of LIN and the LIN/β-CD inclusion complex via different routes are summarized in Table 1. Statistical differences were observed between the area under the concentration curve (AUC: 0.04 ± 0.02 and 90.34 ± 34.36 µg·h/mL) and clearance (CL:5.06 ± 2.27 and 1.27 ± 0.55 L/h/kg) between the groups that received LIN and LIN/β-CD orally, respectively. In addition, Fabs were higher with LIN/β-CD (1.26 ± 0.48 and 1.78 ± 0.37) when compared to LIN and Frel of 19.53 ± 7.42 and 17.84 ± 6.75 for healthy and SHR animals, respectively.

Based on the structural models tested in population pharmacokinetics (PopPK), for the administration of LIN i.v., a 2-compartment model with linear elimination was used. The combined error model was the best to estimate the unexplained residual variability (Table 2). With this structural model, the estimated population values of each parameter were obtained (Table 3). The parameters obtained were volume of distribution (V) of 22.4 L, elimination rate constant (ke) of 0.362 h^−1^, and the distribution microconstants (k12 and k21) between compartments of 0.461 and 1.05 h^−1^, respectively. The correlation of the predicted individual and population plasma concentrations of the LIN with the observed values is shown in the Supplementary File (Supplementary Figure S1).

The 2-compartment structural model with first-order absorption and linear elimination with combined error fitted best the concentration values obtained when LIN was administered orally to the animals. The categorical covariates (complexed linalool administered in healthy rats and complexed linalool administered in SHRs) were added to the model. The PK parameter V and the microconstant k12 were significant for the covariates complexed linalool and complexed linalool in SHR animals, verified by the value *p* ≤ 0.05 in Pearson’s test.

After adding the covariates that showed significance in V and k12 of the base model, the values of log-likelihood (−2LL) and Akaike Information Criteria (AIC) obtained had a significant reduction (Table 4). With this structural model, the estimated population values of each parameter were estimated (Table 5). The typical parameters obtained were ka of 0.226 h^−1^, V of 15.28 L/kg, and ke of 0.163 h^−1^. The correlation of the observed plasma concentrations and the predicted population and individual plasma concentrations with the final model, together with the distribution of residues in relation to the time and plasma concentrations, is shown in (Supplementary Figure S2).

### 2.2. Cardiovascular Effects of Intravenous and Oral Administration of LIN and LIN/β-CD

Following intravenous administration of LIN or the LIN/β-CD inclusion complex in spontaneously hypertensive rats (SHR), distinct cardiovascular effects were observed. LIN induced marked hypotension and bradycardia (%MAP: −56.64 ± 5.05; %HR: −54.54 ± 4.84, n = 5). In contrast, administration of LIN/β-CD also resulted in hypotension (%MAP: −40.00 ± 5.45) but did not significantly affect heart rate (%HR: 18.92 ± 0.67, n = 5) when compared to the vehicle-treated control group (Figure 1). The transient tachycardia observed in the original trace occurred after the hypotensive response and is likely a compensatory reflex mechanism, rather than a direct pharmacological effect of the compound.

**Figure 2:**
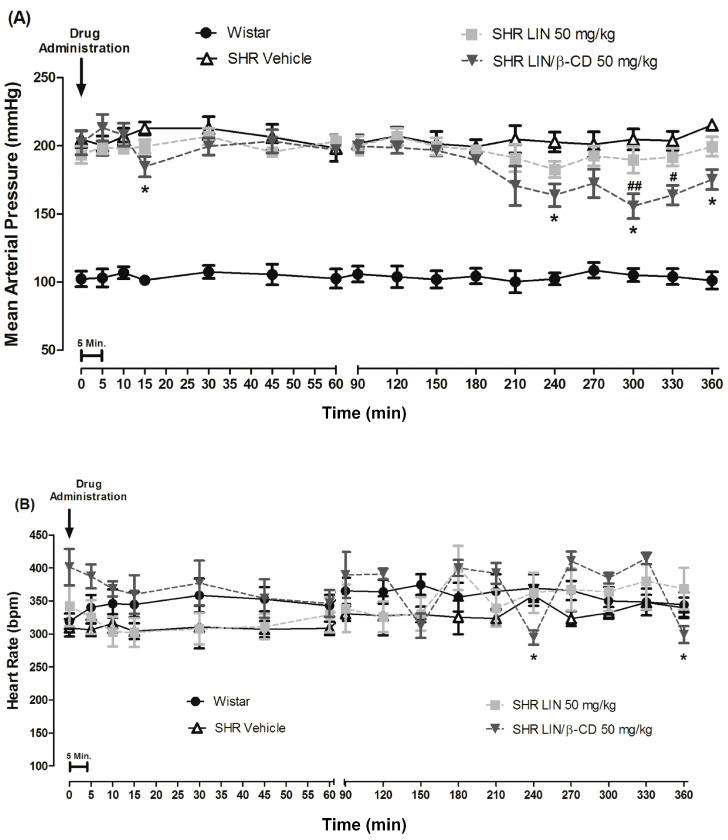
Hypotensive effects following oral administration of LIN/β-CD. (**A**) Line graph illustrating changes in mean arterial pressure (MAP, mmHg) and (**B**) heart rate (HR, bpm) in Wistar and SHRs after orogastric administration of vehicle, LIN, or LIN/β-CD (n = 6). Blood pressure variations were monitored for a 6 h period following administration. Data are expressed as mean ± S.E.M. * *p* < 0.05 vs. SHR Vehicle; # *p* < 0.05 and ## *p* < 0.01 vs. baseline (start of treatment).

Following oral administration of LIN or LIN/β-CD to SHR and normotensive Wistar rats, the LIN/β-CD complex elicited a significant and sustained reduction in BP, with only minor oscillations consistent with expected physiological variability in in vivo measurements. Statistically significant reductions in BP were observed as early as 15 min post-administration and persisted at 4, 5, and 6 h when compared with vehicle-treated animals. Additionally, significant decreases in BP were detected at 5 and 5:30 h relative to baseline values (Figure 2). These results indicate that LIN/β-CD effectively prevented the progression of hypertension in SHR, maintaining BP levels closer to those of normotensive controls, whereas free LIN administered orally failed to produce any significant antihypertensive effect. Furthermore, bradycardic responses were recorded in the LIN/β-CD group at 4 and 6 h post-treatment compared with the vehicle group. Complete datasets are provided in Supplementary Table S1.

### 2.3. Antihypertensive Effects of Chronic LIN and LIN/β-CD Treatment

**Figure 3:**
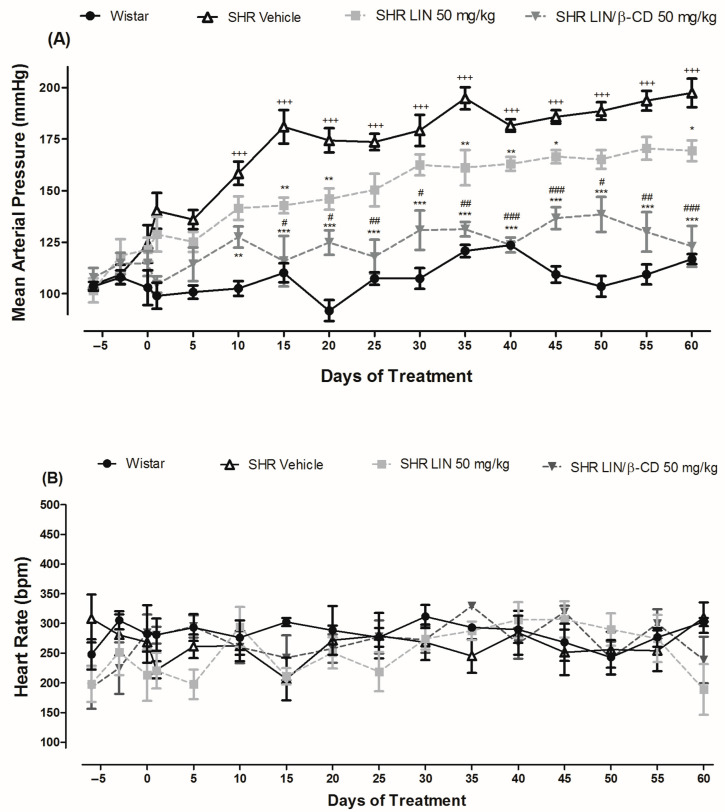
Effect of chronic 60-day LIN/β-CD treatment on blood pressure and heart rate. (**A**) Mean arterial pressure (MAP, mmHg) and (**B**) heart rate (HR, bpm) recorded in Wistar and SHR groups over the 60-day treatment period. Wistar rats (n = 5) received saline, whereas SHR were assigned to Vehicle (n = 5), LIN 50 mg/kg (n = 6), or LIN/β-CD 50 mg/kg (n = 6) treatment groups. All compounds were administered orally once daily. Data are presented as mean ± S.E.M. * *p* < 0.05, ** *p* < 0.01, *** *p* < 0.001 vs. SHR Vehicle; # *p* < 0.05, ## *p* < 0.01, ### *p* < 0.001 vs. SHR LIN; +++ *p* < 0.001 vs. Wistar.

After 60 days of treatment with LIN or LIN/β-CD, no significant changes were observed in the body weight progression of the animals (Supplementary Figure S3), suggesting that the treatments did not interfere with normal growth patterns. However, additional studies would be required to confirm the absence of systemic toxicity. As expected for this hypertensive animal model, vehicle-treated SHRs exhibited significantly elevated mean arterial pressure (MAP = 204.00 ± 20.00 mmHg, n = 5) compared to their normotensive Wistar counterparts (MAP = 116.80 ± 2.41 mmHg, n = 5) throughout the chronic treatment period (Figure 3A).

Treatment with LIN (50 mg/kg) significantly reduced arterial pressure in SHRs, demonstrating a clear antihypertensive effect by attenuating the progression of hypertension when compared to the SHR vehicle group. Notably, chronic treatment with the LIN/β-CD inclusion complex not only reduced BP significantly but also effectively prevented the further development of hypertension in SHRs. The blood pressure levels in this group approached those observed in normotensive controls, suggesting a more sustained antihypertensive effect of the complex (Figure 3A). These results suggest that chronic treatment with LIN/β-CD may enhance its therapeutic performance compared to free LIN, supporting further investigation in long-term hypertension management.

Regarding heart rate parameters, chronic administration of LIN/β-CD did not produce significant alterations, with comparable values observed across all experimental groups (Figure 3B), indicating a selective action on BP without compensatory chronotropic effects.

### 2.4. Evaluation of Vascular Reactivity Following Chronic Treatment

**Figure 4:**
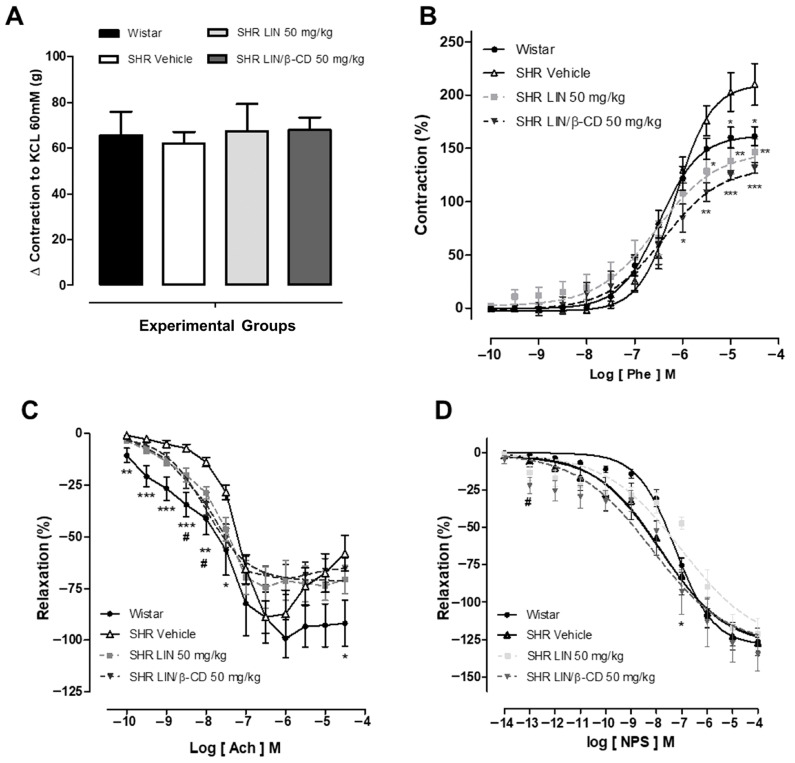
Effect of LIN/β-CD on vascular reactivity of the superior mesenteric arteries. Wistar and SHR animals treated for 60 days. In (**A**) the bar graph demonstrating variations in contraction induced by KCl 60 mM to normalize and equalize the effects of reactivity experiments in wistar (n = 11) treated with saline, or SHR treated with vehicle (n = 7), LIN 50 mg/kg (n = 8) or LIN/β-CD 50 mg/kg (n = 7). In (**B**) Concentration–response curves to phenylephrine (Phe) with * SHR vehicle vs. wistar. In (**C**) Concentration–response curves to acetylcholine (Ach) with * SHR vehicle vs. wistar and # SHR vehicle vs. LIN/β-CD. In (**D**) Concentration–response curves to sodium nitroprusside (SNP) with * SHR LIN vs. SHR LIN/β-CD and # SHR vehicle vs. SHR LIN/β-CD. Concentration–response curves were obtained in isolated superior mesenteric arteries from rats treated according to the following experimental groups: Wistar (n = 6), vehicle (n = 6), LIN 50 mg/kg (n = 7), and LIN/β-CD 50 mg/kg (n = 7). Values expressed as mean ± S.E.M with * *p* < 0.05, ** *p* < 0.01, # *p* < 0.05 and *** *p* < 0.001.

To assess whether chronic treatment induced beneficial alterations in vascular function, vascular reactivity assays were performed using isolated superior mesenteric artery segments. Figure 4 illustrates representative concentration–response curves for phenylephrine (Phe), acetylcholine (ACh), and sodium nitroprusside (SNP), along with the corresponding graphical analyses. Initially, KCl (60 mM)-induced contractions were used as a reference to normalize contractile responses, and no statistically significant differences were observed among the experimental groups, indicating preserved baseline contractile capacity (Figure 4A). Regarding the contractile responses to phenylephrine (10^−10^ to 10^−5^ M), mesenteric artery rings from LIN/β-CD-treated animals exhibited a rightward shift in the concentration–response curve, suggesting reduced vascular reactivity. This effect was statistically significant in terms of maximal efficacy (Emax) when compared across the groups: Wistar (161.52 ± 8.85, n = 11), SHR vehicle (210.16 ± 19.64, n = 8), LIN (146.72 ± 12.34, n = 7), and LIN/β-CD (131.78 ± 5.10, n = 7) (Figure 4B). These results support an antihypertensive vascular adaptation in response to chronic treatment, particularly with the LIN/β-CD complex.

In the endothelium-dependent vasorelaxation assays with acetylcholine, LIN/β-CD treatment also showed a marked vasodilatory response. The Emax values were: Wistar (−91.84 ± 11.14, n = 6), SHR vehicle (−57.96 ± 8.72, n = 8), LIN (−70.52 ± 6.80, n = 7), and LIN/β-CD (−65.29 ± 5.22, n = 8), with the complex group showing significantly improved relaxation compared to the vehicle (Figure 4C). For endothelium-independent relaxation assessed with SNP, only the LIN/β-CD group demonstrated a statistically significant enhancement in vasorelaxation, with Emax values of: Wistar (−126.31 ± 9.24, n = 6), SHR vehicle (−127.39 ± 5.44, n = 7), LIN (−121.04 ± 10.45, n = 6), and LIN/β-CD (−134.92 ± 11.09, n = 8) (Figure 4C). Together, these findings indicate that chronic treatment with LIN/β-CD improves vascular function in hypertensive animals by attenuating vasoconstrictor responses and enhancing both endothelium-dependent and -independent vasorelaxation, supporting its beneficial role in long-term cardiovascular management.

### 2.5. Cardiovascular Morphology and Histological Evaluation

**Figure 5:**
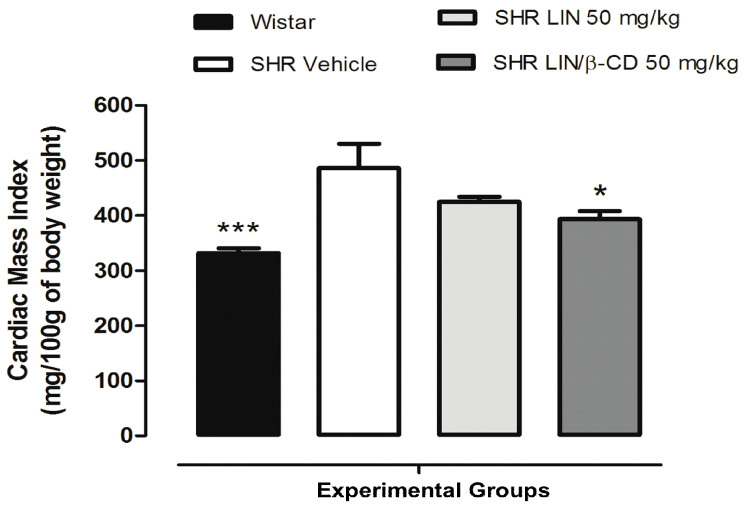
Effect of chronic 60-day LIN/β-CD treatment on the cardiac mass index. Wistar rats (n = 5) received saline, whereas SHR were allocated to the Vehicle (n = 5), LIN 50 mg/kg (n = 6), or LIN/β-CD 50 mg/kg (n = 6) treatment groups. All treatments were administered orally once daily for 60 days. Data are expressed as mean ± S.E.M. * *p* < 0.05, *** *p* < 0.001 vs. SHR Vehicle.

Chronic treatment for 60 days led to a significant reduction in cardiac mass index in SHRs treated with LIN/β-CD compared to the SHR Vehicle group, suggesting a cardioprotective and potential antihypertensive effect of the formulation (Figure 5). As expected, normotensive wistar rats presented lower cardiac mass values, serving as the physiological baseline for comparison.

**Figure 6:**
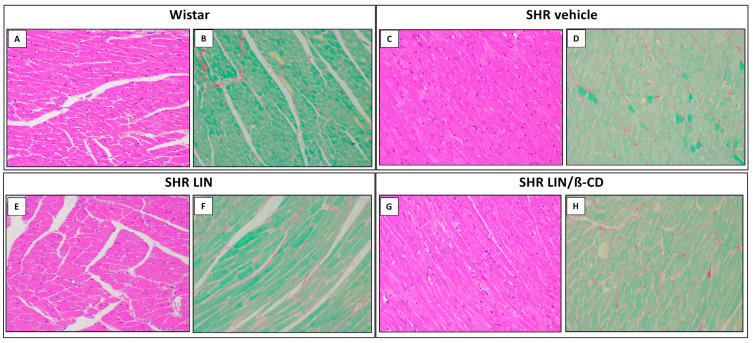
Representative histological sections of cardiac tissue from Wistar and SHR groups. Photomicrographs (200x) show longitudinal sections of the left ventricle stained with hematoxylin–eosin (**A**,**C**,**E**,**G**) and Picrosirius Red (**B**,**D**,**F**,**H**), corresponding, respectively, to Wistar, SHR Vehicle, SHR LIN, and SHR LIN/β-CD groups. HE staining revealed preserved myocardial architecture with organized, well-aligned cardiomyocyte fibers and no morphological features suggestive of hypertrophy or tissue injury. PSR staining confirmed normal collagen distribution and fiber integrity, with no evidence of excessive deposition or structural remodeling across treatment groups.

Histological analysis of the left ventricle revealed preserved myocardial architecture across all groups (Figure 6). Cardiomyocytes exhibited typical morphology, including centrally located ovoid nuclei, striated cytoplasm, and well-defined branching fibers. No evidence of pathological hypertrophy was identified, such as fiber thickening or nuclear polymorphism. Notably, the LIN/β-CD-treated group maintained myocardial structural integrity, comparable to that observed in Wistar controls.

**Figure 7:**
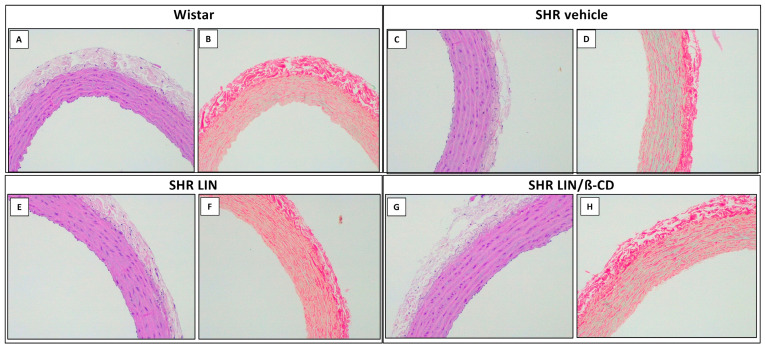
Representative histological sections of aortic tissue from Wistar and SHR groups. Photomicrographs (200x) show longitudinal sections of the aorta stained with hematoxylin–eosin (**A**,**C**,**E**,**G**) and Picrosirius Red (**B**,**D**,**F**,**H**), corresponding, respectively, to Wistar, SHR Vehicle, SHR LIN, and SHR LIN/β-CD groups. HE staining demonstrated preserved aortic wall architecture, with intact intima, media, and adventitia, and no signs of inflammatory infiltrate, intimal thickening, or smooth muscle disorganization. PSR staining revealed normal collagen fiber organization without abnormal accumulation or structural disruption following LIN or LIN/β-CD treatment.

The histological sections of the aorta also showed preserved vascular architecture in all experimental groups (Figure 7). The intimal layer was thin, composed of loose connective tissue lined by an intact endothelium. The medial layer consisted predominantly of elastic fibers interspersed with smooth muscle cells, while the adventitia presented typical vasa vasorum distribution. Importantly, no signs of vascular remodeling were observed, such as intimal thickening, inflammatory infiltrates, medial hyperplasia, or increased collagen deposition, highlighting the vascular safety profile of LIN and LIN/β-CD treatments over the 60-day period.

Microscopic evaluation using hematoxylin-eosin staining demonstrated a healthy myocardium in the wistar group (A), characterized by normal interstitial spacing and absence of cellular hypertrophy. SHR Vehicle animals (C) showed slight reductions in interstitial space and mild disorganization of myocardial fibers. SHRs treated with LIN (E) and LIN/β-CD (G) displayed preserved myocardial morphology with organized, striated cardiomyocytes and centrally located nuclei, consistent with a protective or mitigating effect against hypertensive damage.

Picrosirius Red staining revealed normal collagen distribution in both myocardial and aortic tissues. Mature collagen fibers appeared red, while immature fibers stained green. In wistar (B) and LIN-treated SHR (F) groups, collagen fibers were well-organized, and interstitial space was preserved. The SHR Vehicle group (D) exhibited a subtle increase in red-stained collagen and minor fiber disorganization, whereas the LIN/β-CD group (H) demonstrated a more preserved profile, similar to normotensive controls, suggesting attenuation of extracellular matrix remodeling.

Regarding the aorta, wistar (I) exhibited typical smooth muscle layers with clear organization. SHR Vehicle animals (K) presented mild reduction in interlaminar space and localized cellular clustering, indicating early signs of vascular remodeling. In contrast, both LIN (M) and LIN/β-CD (O) treatment groups maintained normal histological characteristics, including preserved medial layer integrity and smooth muscle alignment. Picrosirius Red staining of the aorta further supported these findings. In Wistar (J), collagen distribution was uniform and well-organized. SHR vehicle sections (L) showed slightly increased red staining and marginal fiber misalignment. However, LIN (N) and LIN/β-CD (P) groups preserved collagen organization with no signs of fibrosis or arterial wall thickening.

Together, these findings suggest that chronic treatment with LIN/β-CD not only reduced cardiac mass index in hypertensive animals but also preserved myocardial and vascular morphology, indicating an effective antihypertensive action without inducing structural cardiac or vascular damage.

## 3. Discussion

In this study, the LIN/β-CD inclusion complex significantly increased systemic linalool bioavailability, leading to improved vascular reactivity, attenuation of cardiac hypertrophy, and enhanced endothelial function in SHR. Histological assessments revealed no signs of cardiac or vascular injury, reinforcing the safety of chronic administration. Collectively, these findings position the LIN/β-CD complex as a promising and well-tolerated candidate for antihypertensive therapy.

Bioavailability is one of the critical determinants of drug therapy efficacy, representing the fraction of an administered dose that successfully reaches systemic circulation. This parameter is intrinsically affected by multiple factors, including the route of administration, the physicochemical properties of the compound, individual patient characteristics, and physiological variables such as baseline BP and metabolic rate [45,46]. Among available options, the oral route remains the most commonly employed for the delivery of antihypertensive agents, largely practicality and greater patient compliance [47,48].

Our findings demonstrated that free LIN, when administered orally, exhibits extremely low systemic absorption, with a bioavailability of approximately 0.003%. However, upon complexation with β-CD, the bioavailability increased markedly to 1.26 ± 0.48%, representing a 20-fold enhancement. Furthermore, the LIN/β-CD complex exhibited a prolonged elimination half-life (t1/2) and reduced clearance (Cl), indicating that β-CD not only protects the active compound but also slows its elimination from the body. This leads to sustained plasma concentrations, thereby extending its therapeutic effects. Tissue distribution analyses further confirmed that LIN/β-CD significantly increased the concentration of LIN in systemic circulation, approximately 20-fold compared to free LIN, enhancing its capacity to reach target cardiovascular tissues effectively.

Additionally, the observed reduction in drug clearance, particularly by renal and hepatic pathways, suggests that LIN complexed with β-CD remains in systemic circulation longer, thereby maximizing its pharmacodynamic effects. Population pharmacokinetic modeling revealed that LIN/β-CD altered key pharmacokinetic parameters, including distribution volume and elimination constants, in a manner that improved the drug’s dispersion across organs and tissues. This resulted in a greater number of LIN molecules remaining available in the bloodstream, a pharmacologically advantageous outcome.

Overall, both individual and population-based pharmacokinetic analyses demonstrate that complexation with β-CD significantly enhances LIN ability to permeate tissues and exert sustained biological effects, with diminished susceptibility to rapid renal or hepatic elimination [46]. These findings suggest that LIN/β-CD is substantially more effective in exerting cardiovascular effects compared to free LIN. Notably, no significant differences in pharmacokinetic behavior were observed between normotensive and SHR animals, reinforcing the robustness of our results.

Study has investigated the oral administration of LIN for cardiovascular effects [19], it is noteworthy that a 200 mg dose of pure LIN was required to elicit a maximal hypotensive response three hours post-administration. In contrast, our study employed a significantly lower dose, only 50 mg of LIN, either in its free form or complexed with β-CD, administered via the same oral route. Remarkably, despite using just a quarter of the previously tested dose, we observed a statistically significant reduction in BP only in the group treated with the LIN/β-CD complex. This effect was evident when comparing systolic BP over time following drug administration, and became statistically significant as early as 15 min, as well as at the 4th, 5th, and 6th hours post-treatment, in order to highlight the enhanced and sustained hypotensive action associated with the complex. Furthermore, animals receiving LIN/β-CD maintained BP levels that closely approximated those of normotensive controls throughout the experimental period, effectively preventing the progressive hypertensive profile typically observed in SHR animals.

These findings suggest a superior antihypertensive and blood pressure–modulating potential of the LIN/β-CD complex when compared to free LIN and vehicle-treated SHR controls. Hypertension is widely recognized to induce significant structural and functional alterations in the endothelium and vascular smooth muscle cells, primarily due to sustained hemodynamic stress and chronic inflammation [49,50,51,52,53]. Such changes are associated with endothelial dysfunction, characterized by impaired nitric oxide (NO) synthesis, decreased NO bioavailability, and disruption of downstream signaling pathways [54,55,56]. These impairments compromise vascular homeostasis and contribute to the progression of hypertension. Previous studies have reported that racemic LIN promotes vasorelaxation through both endothelium-independent and endothelium-dependent mechanisms, depending on the vascular bed studied. LIN induced endothelium-independent relaxation in rat mesenteric arteries [19], while in mouse aorta, relaxation was found to be endothelium-dependent, involving NO release, soluble guanylyl cyclase activation, and potassium channel opening [36].

In our study, chronic oral administration of the LIN/β-CD inclusion complex for 60 days significantly attenuated Phe-induced vasoconstriction in mesenteric rings from SHR. This reduction in vascular reactivity was more pronounced than that observed with free LIN and suggests an enhanced vasoprotective effect associated with the complexed formulation. These findings point to a modulatory effect of LIN/β-CD on vascular tone, which may contribute to its long-term antihypertensive potential.

Moreover, experiments using SNP, a classic NO donor, revealed a leftward shift in the concentration–response curve in the LIN/β-CD-treated group compared to both the vehicle and LIN-treated groups, indicating increased vascular sensitivity to NO. This enhanced responsiveness suggests that treatment with LIN/β-CD may restore or potentiate NO signaling pathways, often impaired in hypertension. Similarly, evaluation of endothelium-dependent relaxation through cumulative administration of ACh showed that the LIN/β-CD group exhibited a significant leftward shift in the concentration–response curve. This indicates improved endothelial function and reinforces the superior efficacy of the inclusion complex in enhancing NO-mediated vasorelaxation when compared to free LIN and vehicle-treated SHR animals. Notably, the vasorelaxant responses in the LIN/β-CD group approached those observed in normotensive Wistar rats, highlighting the restorative potential of this formulation.

In hypertensive conditions, NO signaling is frequently disrupted not only due to impaired eNOS activity and L-arginine deficiency but also by oxidative degradation and impaired activation of downstream effectors such as protein kinase G (PKG) [15]. In this context, our findings demonstrate that chronic administration of the LIN/β-CD inclusion complex significantly enhances NO signaling and endothelial function in SHR. These improvements suggest that the complex mitigates fundamental pathophysiological mechanisms underlying hypertension. By promoting vascular relaxation and restoring endothelial responsiveness, LIN/β-CD emerges as a promising therapeutic strategy capable of addressing the molecular basis of endothelial dysfunction, thus offering translational potential for the management of cardiovascular diseases associated with NO deficiency.

In agreement with the literature, our data demonstrated that normotensive Wistar rats exhibited significantly lower cardiac mass index compared to vehicle-treated SHR, confirming the characteristic cardiac remodeling observed in hypertensive animals. Interestingly, when analyzing the effects of chronic treatment, only the LIN/β-CD-treated group showed a statistically significant reduction in cardiac mass index among the hypertensive animals. This reduction was not observed in the group treated with free LIN. Histological evaluation of cardiac tissue further supported the safety of the treatment, because do not have morphological alterations indicative of pathological remodeling were observed in any group. The cardiac tissue presented normal architecture, with striated cytoplasm, centrally located oval nuclei, and regular myocyte branching patterns. Importantly, none of the groups, including those treated with LIN or LIN/β-CD, exhibited histopathological signs of cardiac hypertrophy, such as myocyte disarray, nuclear atypia, or interstitial fibrosis.

Similarly, analysis of aortic tissue using hematoxylin–eosin and picrosirius red staining revealed no fibrous thickening of the intimal layer, inflammatory infiltrates, or collagen deposition in any of the groups, including SHR. Our findings indicate that chronic treatment with the LIN/β-CD inclusion complex significantly attenuates cardiac remodeling in SHR, as evidenced by a reduction in the cardiac mass index. This suggests a protective effect of the complex against hypertrophy progression. By mitigating key contributors such as oxidative stress and endothelial dysfunction, LIN/β-CD not only preserves cardiac structure but may also support improved functional outcomes.

These benefits, combined with the absence of histological damage, underscore the therapeutic potential of the complex in preventing or delaying hypertensive cardiomyopathy. Taken together, the histopathological and morphometric findings reinforce the beneficial role of LIN/β-CD in cardiovascular modulation. While both LIN and LIN/β-CD were evaluated, only the inclusion complex significantly reduced cardiac mass index without inducing structural cardiac or vascular alterations.

The important limitation of this study is the lack of comparison with clinically established antihypertensive drugs. Although previous work from our group demonstrated the antihypertensive activity of free linalool compared to captopril [32], the present study did not assess the LIN/β-CD complex against conventional therapies. These findings suggest that LIN/β-CD represents a promising experimental strategy for blood pressure modulation. However, further comparative studies with standard antihypertensive drugs are required to establish its clinical applicability.

These results suggest that LIN/β-CD may not only exert superior antihypertensive effects compared to free LIN but also contribute to the attenuation of hypertension-induced cardiac remodeling. In summary, the in vivo and in vitro data presented here demonstrate that chronic administration of LIN/β-CD results in greater cardiovascular protection in SHR animals than LIN alone. These findings support the potential of the LIN/β-CD inclusion complex as a promising therapeutic strategy for the management of hypertension and its associated cardiac complications. Further studies are warranted to elucidate the molecular mechanisms underlying these beneficial effects and to confirm its translational applicability.

## 4. Material and Methods

### 4.1. Material

All reagents were obtained from Sigma-Aldrich (St. Louis, MO, USA), including sodium nitroprusside, (-)-phenylephrine, acetylcholine chloride, captopril, cremophor, Tween 20, aprotinin A, potassium iodide (KI), benzethonium chloride, EDTA, β-cyclodextrin (β-CD), and (-)-linalool (purity ≥ 95.0%). For vehicle preparation, cremophor (0.003 g/mL) was solubilized in 0.9% NaCl (for in vivo protocols) or distilled water (for in vitro assays). The monoterpene (-)-linalool was dissolved in cremophor at a 2:1 ratio (LIN/cremophor).

The LIN/β-CD inclusion complex can be prepared using three classical methods: (i) physical mixture—manual mixing of LIN with powdered β-CD in a 1:1 molar ratio; (ii) paste complexation—homogenization of β-CD (1135 mg) with water (1.2:4, *w*/*v*), followed by the addition of 154 mg of LIN and manual agitation, with subsequent air drying and storage; and (iii) slurry complexation—stirring of LIN (154 mg) and β-CD (1135 mg) in water (3:4, *w*/*v*) for 36 h, followed by drying as previously described [48]. However, the formulation used in the pharmacological assays was the slurry complex, selected because it provides superior complexation yield and stability. Moreover, the slurry method affords higher practical yield and generates a more homogeneous solid phase, making it particularly suitable for pharmacological applications.

Tyrode’s solution used in the ex vivo assays was prepared using NaCl (158.3 mM), NaHCO_3_ (10.0 mM), NaH_2_PO_4_ (0.42 mM), KCl (4.0 mM), MgCl_2_ (1.05 mM), CaCl_2_·2H_2_O (2.0 mM), and glucose (5.6 mM), obtained from Sigma (Sigma-Aldrich, St. Louis, MO, USA) or Vetec (Vetec, Rio de Janeiro, Brazil).

### 4.2. Quantification of LIN in Rat Plasma Samples

A bioanalytical methodology was developed and validated to quantify LIN in rat plasma using high-performance liquid chromatography with UV detection (HPLC/UV), according to RDC 27/2012 of the National Agency of Sanitary Vigilance, Brazil [57]. Plasma aliquots (100 µL) were extracted with 300 µL of acetonitrile.

The samples were vortexed for 3 min and centrifuged at 13,000× *g* for 15 min, at 8 °C. The supernatant aliquots were injected into an HPLC instrument (PerkinElmer, Waltham, MA, USA), Flexar™ HPLC model), using reversed-phase column C18 (Nucleosil column 250 mm × 4.6 mm internal diameter, particle size 5 μm (Macherey—Nagel, Versmold, Germany) protected by a guard column with the same material (Hypersil BDS, 10 mm, particle size 5 μm (Thermo Scientific, Waltham, MA, USA) at a temperature of 50 ± 1 °C during the analysis.

The mobile phase was composed of water: acetonitrile (70:30, *v*/*v*), at a flow rate of 1 mL/min. LIN was detected at a wavelength of 210 nm, and the peak area was used for the quantitation of samples. The method was precise, sensitive and accurate within the concentration range of 5–100 ng/mL. All samples quantified were between the concentration range, the higher concentrations were diluted to reach the higher linear concentration.

### 4.3. Pharmacokinetics Analysis

Plasma pharmacokinetic parameters were estimated after administration of 50 mg/kg and 100 mg/kg, i.v. and orally, respectively, of LIN; 100 mg/kg orally of LIN/β-CD, in healthy Wistar rats; and 50 mg/kg of LIN/β-CD, in SHRs.

The animals received the drugs at the above doses through the caudal lateral vein or by oral gavage. Six animals were evaluated per group, as stated in the literature [45,46]. After administration of the compounds, approximately 200 µL of blood samples were collected from the caudal lateral vein at 0.083, 0.25, 0.5, 1, 1.5, 2, 4, 6, and 8 h. Plasma was separated by centrifugation (6800× *g* at 8 ± 1 °C for 15 min) and stored at −20 °C until analysis. Non-compartmental analysis (NCA) of the individual concentration-time profiles was performed using Excel^®^ 2016 software (Microsoft Corporation, Redmond, DC, USA).

Calculated pharmacokinetic parameters included: the elimination rate constant (ke), absorption rate constant (ka), area under the concentration-time curve from 0 h to infinity (AUC_0−∞_), clearance (Cltot), half-life (t1/2), the volume of distribution (V), mean residence time (MRT), mean absorption time (MAT), absolute bioavailability (Fabs) and relative bioavailability (Frel). All the pharmacokinetic (PK) parameters were estimated individually aby analyzing the individual PK profiles. Results were expressed as the mean ± SD, and the PK parameters were compared by ANOVA followed by Student’s *t*-test (α = 0.05).

Population pharmacokinetic analysis was performed using Monolix^®^ Suite 2019R1 software (Lixoft^®^, Antony, France). Structural models evaluated included 1- and 2-compartment models, first-order absorption, and linear elimination. Different error models were analyzed to explain the variability of the residual error, including a constant, proportional, and combined error model. These data were described using a log-normal distribution. The models were parameterized in V, ka, ke, and distribution constants (k1-2 and k2-1). The parameters were estimated with a stochastic approximation. The object function value (OFV), represented as −2 times the log-likelihood (−2LL) and Akaike Information Criterion (AIC), was used to compare the models.

The base model for i.v. and oral administration has been established. For oral linalool, after establishing the base model, new models adding the categorical covariates investigated in the study (complexed linalool administered in healthy rats and complexed linalool administered in SHRs) were developed. The visual analysis of the graphs and statistical tests (Pearson’s correlation and ANOVA) were carried out to verify if there is any relationship between the random effects of the parameters (h) and the covariables that could contribute to improving the model. The addition of the covariate was considered statistically significant when it resulted in a significant reduction in the value of −2LL (c2, *p* < 0.05, degrees of freedom = 1) and of AIC.

The graphs that correlate the plasma concentrations with the predicted individual or population plasma concentrations were evaluated by individual waste distribution charts (IWRES). Also, to evaluate whether simulations performed from the developed model are capable of reproducing the median and the variability of the observed data, a graph was generated to verify the predicted data visual predictive check (VPC).

### 4.4. LIN and LIN/β-CD Acute Effects at Blood Pressure and Heart Rate

This protocol aimed to evaluate the acute hemodynamic effects of LIN, administered intravenously (i.v.), on blood pressure (BP) and heart rate (HR) in spontaneously hypertensive rats (SHR) and normotensive Wistar rats. Animals were anesthetized with ketamine and xylazine (Cristália), and following trichotomy of the iliac region, polyethylene (PE) catheters were implanted into the femoral artery and vein.

The catheters consisted of PE-10 segments (internal/external diameter: 0.28/0.61 mm) connected to PE-50 catheter (internal/external diameter: 0.58/0.96 mm). To prevent catheter blockage during the hemodynamic recordings, the arterial and venous catheters were implanted pre-filled with a sterile saline–heparin solution (heparin: 100 UI). After catheterization, the catheters were exteriorized subcutaneously to the dorsal cervical region and suture from the initial incision was made. Postoperative care included administration of a veterinary pentabiotic solution (0.2 mL; Fort Dodge) and a single dose of the analgesic and anti-inflammatory drug flunixin meglumine (0.1 mL; Banamine^®^, Schering-Plough Animal Health, Whitehouse Station, NJ, USA) to ensure animal comfort and prevent infection or inflammation.

Following a 24 h recovery period, baseline physiological parameters, including systolic BP (SBP), diastolic BP (DBP), and heart rate, were recorded. BP was continuously monitored via the arterial catheter connected to a pressure transducer (ADInstruments, São Paulo, Brazil). The signal was amplified and digitized at 1 kHz using a PowerLab 8/35 data acquisition system and analyzed with LabChart 8 Pro software (ADInstruments, São Paulo, Brazil). Once cardiovascular parameters stabilized, SBP, DBP, and HR were recorded as baseline control values. To confirm catheter patency and vascular responsiveness, sodium nitroprusside (SNP) was administered intravenously to induce transient hypotension. After BP returned to baseline, intravenous (i.v.) and oral administrations of LIN or LIN/β-CD (50 mg/kg) were initiated according to the experimental design.

### 4.5. Chronic Effects of LIN and LIN/β-CD on Cardiovascular Parameters

Before starting any treatment, animals were conditioned for one week to minimize stress-related variability in BP and HR measurements. SHRs were randomly assigned to three experimental groups: Group 1 (SHR + Vehicle): Received saline solution (0.1 mL/100 g, p.o.); Group 2 (SHR + LIN): Treated with (-)-linalool (50 mg/kg/day, p.o.); and Group 3 (SHR + LIN/β-CD): Treated with the LIN/β-cyclodextrin inclusion complex (50 mg/kg/day, p.o.). An additional control group of normotensive Wistar rats (Group 4: Wistar + Vehicle) received saline under the same dosing protocol to serve as a baseline.

All treatments were administered orally once daily (between 08:00 and 12:00 h) for a period of 60 days, and cardiovascular assessments were performed with all groups in the afternoon (between 12:00 and 18:00 h), approximately 60 min post-administration. BP and heart rate were measured every five days throughout the treatment period using the tail-cuff plethysmography method (Insight^®^ V2.11, São Paulo, Brazil). Systolic and diastolic blood pressures were measured in triplicate for each animal, and the average of these values was used for data analysis and graphical representation.

### 4.6. Chronic Effects of LIN and LIN/β-CD on Vascular Reactivity

Upon completion of the 60-day treatment period, vascular reactivity was assessed using isolated superior mesenteric artery rings obtained from SHRs (200–250 g), as previously described [30]. The arteries were excised, carefully cleaned of connective tissue and adipose deposits, and sectioned into 2 mm rings. These rings were mounted in organ baths containing oxygenated Tyrode’s solution (95% O_2_ and 5% CO_2_) maintained at 37 °C. Each arterial ring was set to an optimal resting tension of 0.75 g, as previously determined, and allowed to equilibrate for 60 min. During this stabilization period, tension was periodically adjusted to maintain baseline force. Isometric contractions were recorded using a force transducer (FORT-10; WPI, Sarasota, FL, USA) connected to an amplifier system (Miobath-4; WPI, Sarasota, USA), with data digitized via an analog-to-digital converter and stored on a personal computer.

Endothelial integrity was verified by assessing relaxation responses to acetylcholine (ACh, 10^−6^ M) following phenylephrine (Phe, 1 µM)-induced contraction. Rings exhibiting <10% relaxation were considered endothelium-denuded, while those showing >60% relaxation were classified as endothelium-intact. After washout, rings were re-contracted with Phe (10^−6^ M) to generate consistent pre-contraction levels. Depolarizing KCl solution (60 mM) was applied to assess maximal contractile responsiveness. Concentration–response curves for Phe (10^−10^ to 10^−5^ M) were expressed as a percentage of the maximal contraction induced by KCl solution. Conversely, relaxation responses to sodium nitroprusside (SNP, 10^−13^ to 10^−5^ M) were expressed as a percentage of the relaxation relative to the maximal Phe-induced contraction (10^−6^ M).

### 4.7. Effects of LIN and LIN/β-CD on Cardiac Mass Index and Histopathological Morphophysiology

After 60 days of treatment period, Wistar and SHRs were euthanized in a carbon dioxide (CO_2_) chamber. Hearts were carefully excised, and surrounding adipose tissue and vascular structures were meticulously removed. Each heart was weighed using an analytical balance, and the cardiac mass index was calculated by normalizing the heart weight to the total body weight of the animal. A transverse cut was then made, and the left and right ventricles, and thoracic aortas. Both tissues were fixed in 4% formaldehyde for histological processing.

The hearts and thoracic aortas were fixed in 4% buffered formaldehyde (0.1 M sodium phosphate buffer, pH 7.4), followed by washing in distilled water. Tissue dehydration was carried out in a graded ethanol series of increasing concentrations, ending with absolute ethanol. Samples were then cleared in xylene, embedded in paraffin, and sectioned using a microtome (RM2125RT, Leica^®^, Wetzlar, Germany) to obtain serial 5 μm sections. Tissue sections were mounted on glass slides and stained with hematoxylin-eosin (HE) for general histological evaluation. Additional sections were stained with Picrosirius Red, which selectively stains collagen and enables qualitative assessment of connective tissue, in order to compare and separate type I and type III collagen fibers.

Histological evaluation was conducted in a blinded manner. The descriptive analysis focused on sections from the aorta and both left and right ventricular myocardium. Parameters assessed included the presence or absence of inflammatory infiltrates, hemorrhagic areas, and structural alterations such as decreased cardiomyocyte volume, disruption of muscle fiber continuity, loss of transverse striations, sarcoplasmic granulation, contraction bands, and nuclear changes indicative of tissue injury. Histological images were captured using a digital camera system (DP71, Olympus, Tokyo, Japan) coupled to a light microscope (BX-51, Olympus, Japan). Image acquisition and processing were performed using a laptop (Dell, Intel^®^ Core™ i5) and Analysis Get It 2.2 software (Olympus).

### 4.8. Statistical Analysis

Data are expressed as mean ± standard error of the mean (SEM). Concentration–response curves were generated using nonlinear regression analysis to determine the maximal response (Emax) and the concentration required to elicit 50% of the maximal effect (EC50). Statistical comparisons between groups were made using either Student’s *t*-test or one-way analysis of variance (ANOVA), followed by the Bonferroni post hoc test when appropriate. A *p*-value of less than 0.05 was considered statistically significant. All statistical analyses were performed using GraphPad Prism^®^ version 5.0 (GraphPad Software Inc., San Diego, CA, USA).

## 5. Conclusions

This study provides compelling evidence that chronic administration of the LIN/β-CD inclusion complex elicits pronounced antihypertensive effects in SHRs. The complex significantly improved vascular reactivity, enhanced endothelial function, and reduced the cardiac mass index-key parameters associated with hypertension-induced cardiovascular damage. Notably, LIN/β-CD demonstrated superior efficacy compared to free linalool, underscoring the role of β-cyclodextrin in enhancing the bioavailability and therapeutic activity of linalool.

Histopathological analyses confirmed the absence of cardiac or vascular toxicity, supporting the long-term safety profile of the complex. Collectively, these findings position LIN/β-CD as a promising, well-tolerated, and naturally derived therapeutic strategy for the management of hypertension and its cardiovascular complications.

From a translational standpoint, this formulation represents a viable candidate for the development of innovative oral antihypertensive therapies, with potential applicability to other conditions involving endothelial dysfunction and vascular remodeling. Further preclinical investigations and clinical validation are warranted to fully elucidate its therapeutic scope and long-term benefits.

## Figures and Tables

**Table 1:** LIN and LIN/β-CD plasma pharmacokinetic parameters calculated by Non-Compartmental Analysis (NCA) after i.v. and oral administration to Wistar rats.

Pharmacokinetic Parameters	LIN Free i.v. 50 mg/kg	LIN Free Oral 100 mg/kg	LIN/β-CD Oral 100 mg/kg	LIN/β-CD Oral SHR 50 mg/kg
ke (h^−1^)	0.13 ± 0.06	0.09 ± 0.06	0.20 ± 0.09	0.13 ± 0.07
t1/2 (h)	6.60 ± 4.04	8.64 ± 4.44	12.08 ± 4.27	8.95 ± 3.52
AUC_0−∞_ (µg·h/mL)	25.28 ± 18.65	0.04 ± 0.02	90.34 ± 34.36 ^a^	51.28 ± 19.29 ^a^
MRT (h)	8.75 ± 4.78	1.97 ± 0.65	2.20 ± 0.64	2.76 ± 0.23
MAT (h)	-	4.69 ± 1.50	6.28 ± 0.64	5.73 ± 0.53
ka (h^−1^)	-	0.23 ± 0.07	0.16 ± 0.01	0.18 ± 0.02
CL (L/h/kg)	3.03 ± 1.67	5.06 ± 2.27	1.27 ± 0.55 ^a^	1.08 ± 0.62 ^a^
Vd (L/kg)	25.19 ± 10.40	16.29 ± 10.73	17.57 ± 8.84	17.9 ± 4.47
Fabs (%)	-	0.003 ± 0.001	1.26 ± 0.48	1.78 ± 0.37
Frel (%)	-	-	19.53 ± 7.42	17.84 ± 6.75

Note. ke, elimination rate constant; t1/2, half-life; AUC_0−∞_, Total Area Under the Curve; MRT, Mean Residence Time; MAT, Mean absorption time; ka, absorption rate constant; CL, clearance; Vd, Volume of Distribution; Fabs, Absolute bioavailability; Frel, Relative Bioavailability. Values are represented as mean ± standard deviation, respectively (n = 6/group). ^a^. Statistically different from LIN free oral (α < 0.05).

**Table 2:** Evaluation of model fit using likelihood and Akaike Information Criterion (AIC) for the two-compartment model.

	Constant	Proportional	Combined
−2 × log-likelihood (−2LL)	28.51	28.87	**26.39**
Akaike Information Criteria (AIC)	46.51	46.87	**46.39**

**Table 3:** Parameter estimates of the LIN population PK model after intravenous administration in healthy rats.

Parameter	Estimate	SE	RSE (%)
V (L)	22.4	4.24	19
ke (h^−1^)	0.362	0.106	29.2
k12 (h^−1^)	0.461	0.209	45.3
k21 (h^−1^)	1.05	0.670	63.8
Residual variability			
a	0.0817	0.0061	7.46
b	2.37	0.11	4.64

Note. V: volume of distribution; ke: first-order elimination rate; k12, k21: distribution constants; SE: Standard error; RSE: Relative standard error.

**Table 4:** Likelihood and Akaike information criteria after adding covariates.

Covariates	−2LL	Difference of −2LL	AIC	Difference in AIC
Base model	617.08		631.08	
Adding Complex, Complex SHR in V and k12	425.04	−192.04	457.04	−174.04

Note. V: volume of distribution; k12: distribution microconstant; log-likelihood (−2LL) and Akaike Information Criteria (AIC).

**Table 5:** Parameter estimates of the LIN population PK model after adding the covariates to the oral administration data.

Fixed Effects	Estimated	SE	RSE (%)
ka (h^−1^)	0.226	0.056	24.9
V (L)	15.28	4.478	29.3
ke (h^−1^)	0.163	0.087	53.4
k12 (h^−1^)	3.37 × 10^2^	6.670	19.8
k21 (h^−1^)	0.0711	0.037	52.0
Residual variability			
a	0.121	0.007	5.78
b	2.93	0.10	3.41

Note. ka: absorption rate constant; V: volume of distribution; ke: first-order rate elimination; k12, k21: distribution microconstants; SE: Standard error; RSE: Relative standard error.

## Data Availability

The original contributions presented in this study are included in the article/Supplementary Materials. Further inquiries can be directed to the corresponding author.

## Supplementary Materials

The following supporting information can be downloaded at https://www.mdpi.com/article/10.3390/ph19010037/s1, Figure S1: Final PopPK model evaluation of intravenous administration in healthy rats. Figure S2: Final PopPK model evaluation of oral administration data. Figure S3: Mean (±SD) linalool (LIN) plasma concentration curves after its administration to Wistar rats (n = 6/group). Table S1: Oral administration effects of LIN e LIN/β-CD at cardiovascular parameters of wistar and SHR. Figure S4: Effect of chronic 60-day treatment with LIN/β-CD on body weight gain. Body weight gain of treated Wistar and SHR animals. Groups Wistar (n = 5), Vehicle (n = 5), SHR LIN 50 mg (n = 6) and SHR LIN/β-CD (n = 6). Values expressed as mean ± S.E.M.
