# Supplementary material for: Complexed Linalool with Beta-Cyclodextrin Improve Antihypertensive Activity: Pharmacokinetic and Pharmacodynamic Insights
Source: Pharmaceuticals (Basel). 2025 Dec 23;19(1):37. doi: 10.3390/ph19010037 (PMC12844744; doi:10.3390/ph19010037)
Supplement: Supplementary file 1 [file pharmaceuticals-19-00037-s001.zip › pharmaceuticals-3974980-supplementary.pdf]

SUPPLEMENTARY FILE

# Complexed Linalool with Beta-Cyclodextrin Improve Antihypertensive Activity: Pharmacokinetic and Pharmacodynamic Insights

**Samuel Camargo [1,2], Carla Medeiros [3], Liliane Silva [2], Rafael Leonne Jesus [2], Fênix Araujo [1], Daniele Brito [1,2], Quiara Alves [1,2], Raiana Moraes [1], Valdeene Santos [4], Francine Azeredo [4,5], Adriano Araújo [6], Lucindo Quintans-Júnior [6] and Darizy Silva [1,2,7,*]**

[1] Gonçalo Moniz Institute, Oswaldo Cruz Foundation (FIOCRUZ), Salvador 41745-715, Brazil; camargo.fisio2016@gmail.com (S.C.); fenixaaraujo@gmail.com (F.A.); danielesb20@gmail.com (D.B.); quiara.lovatti@gmail.com (Q.A.); rai.pharma@hotmail.com (R.M.)
[2] Laboratory of Cardiovascular Physiology and Pharmacology, Federal University of Bahia, Salvador 40110-902, Brazil; barret.liliane@gmail.com (L.S.); rafaelleonne@gmail.com (R.L.J.)
[3] Department of Pharmacology, Ribeirao Preto Medical School, Federal University of São Paulo, São Paulo 14049-900, Brazil; fiamaazevedo19@gmail.com
[4] Laboratory of Pharmacokinetic and Pharmacometrics, Federal University of Bahia, Salvador 40170-115, Brazil; enevieira@hotmail.com (V.S.); francinej@gmail.com (F.A.)
[5] Center for Pharmacometrics and Systems Pharmacology, Department of Pharmaceutics, College of Pharmacy, University of Florida, Orlando, FL 32827, USA
[6] Graduate Program in Health Sciences, Federal University of Sergipe, Aracaju 49060-108, Brazil; adriasa2001@yahoo.com.br (A.A.); lucindojr@gmail.com (L.Q.-J.)
[7] Department of Bioregulation, Federal University of Bahia, Salvador 40110-902, Brazil
* Correspondence: darizy@gmail.com or darizy.silva@ufba.br

**Pharmacokinetics analysis**

**Supplementary Figure S1.** Final PopPK model evaluation of intravenous administration in healthy rats. Goodness-of-fit plots: Observed LIN plasma concentrations vs population (A) and individual (B) predicted concentrations. Population and individual weighted residues IWRES vs time (C) and LIN plasma concentrations (D).

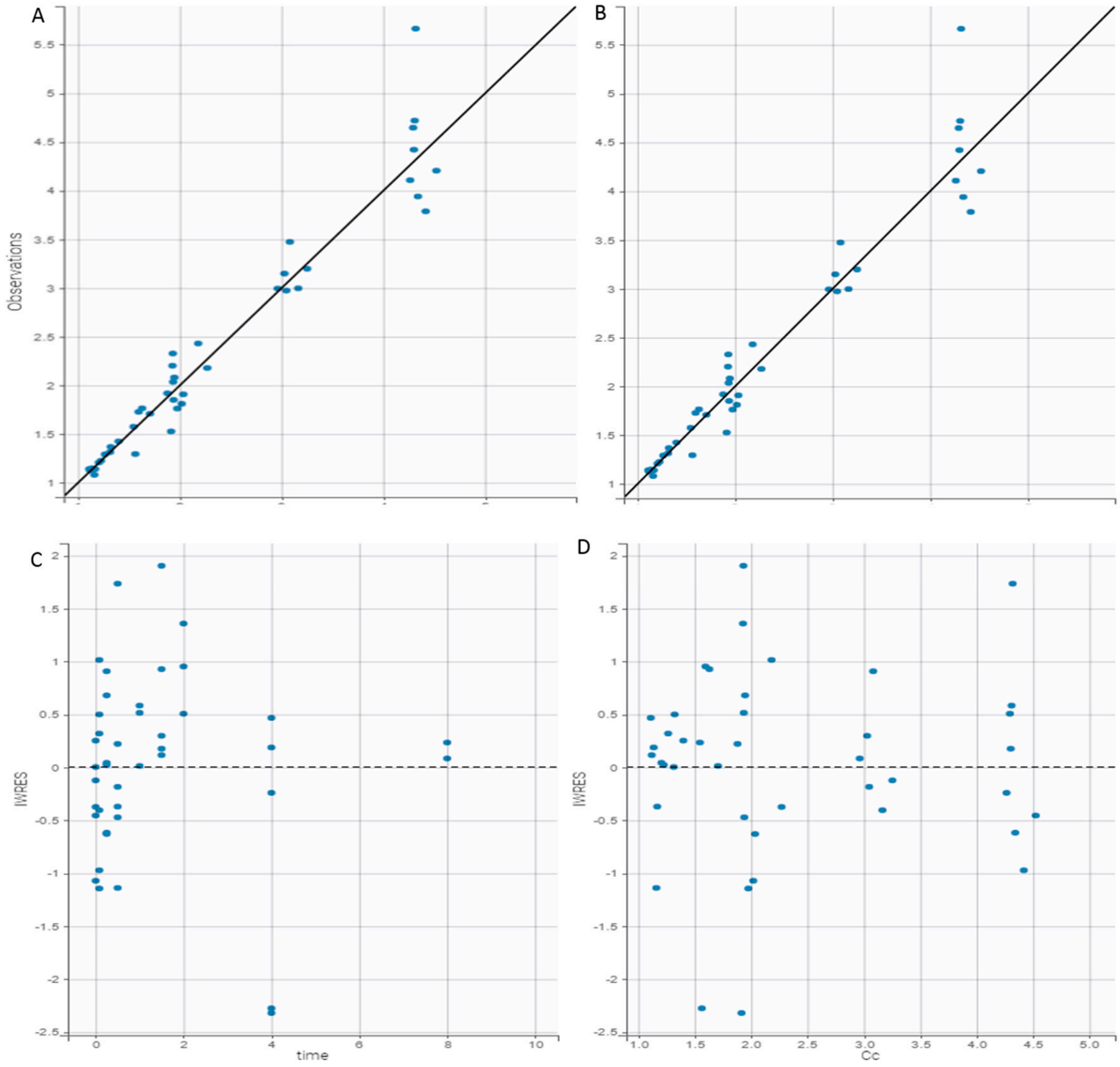

**Supplementary Figure S2.** Final PopPK model evaluation of oral administration data. (A) Goodness-of-fit plots: Observed LIN plasma concentrations vs population (A) and individual (B) predicted concentrations. (B) Population and individual weighted residues (IWRES) vs time (C) and LIN plasma concentrations (D).

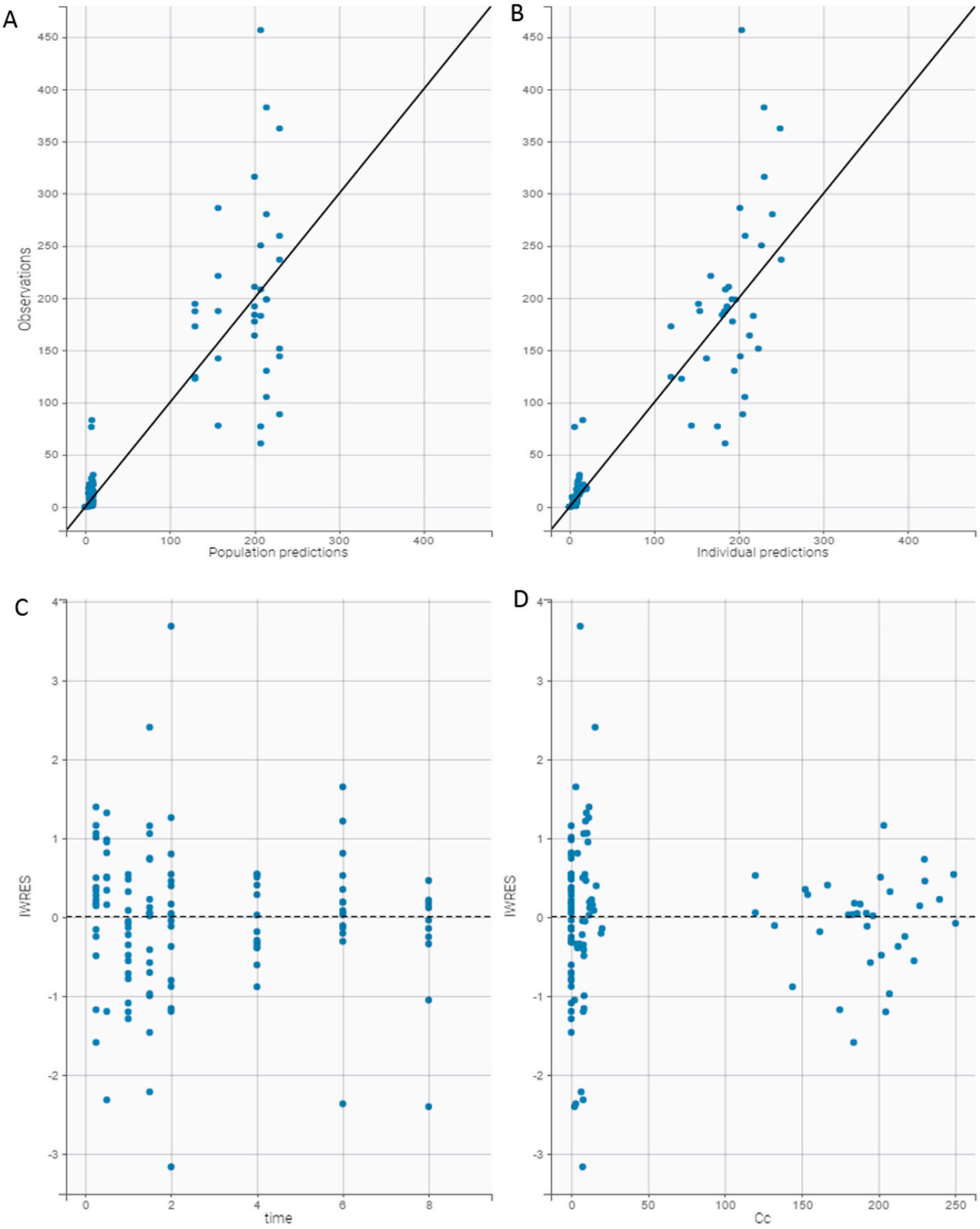

**Supplementary Figure S3:** Mean (±SD) linalool (LIN) plasma concentration curves after its administration to Wistar rats (n = 6/group). LIN free drug intravenously administered at a 50 mg/kg dose (A); LIN free drug orally administered at a 100 mg/kg dose (B); LIN/β-CD orally administered at a 100 mg/kg dose (C).

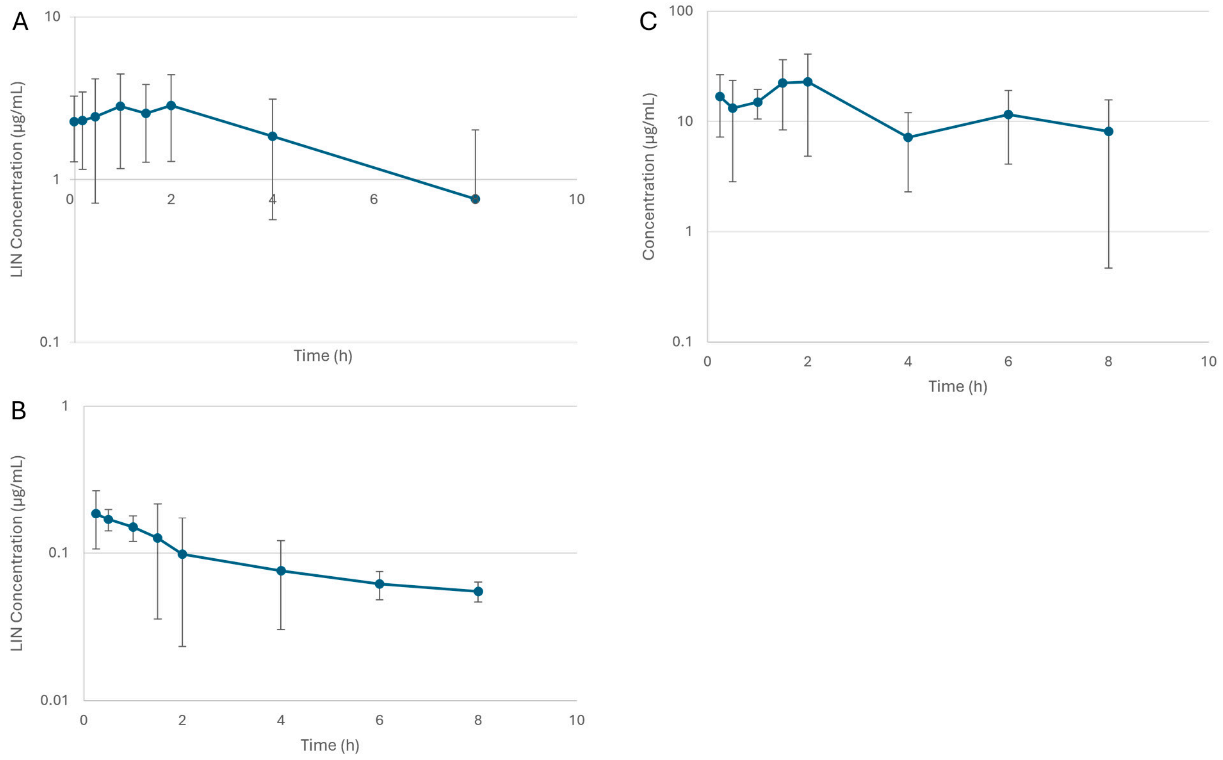

**Cardiovascular Effects of Intravenous and Oral Administration of LIN and LIN/β-CD**

| Groups | Administration Time = Zero | 15 min. | 4 hours | 5 hours | 6 hours |
|---|---|---|---|---|---|
| | | | **Mean Arterial Pressure (MAP)** | | |
| Wistar | 102.28 ± 5.69 | 101.40 ± 3.57 | 100.23 ± 8.16 | 108.64 ± 5.59 | 101.15 ± 6.20 |
| SHR vehicle | 204.86 ± 6.18 | 212.62 ± 4.77 | 204.71 ± 10.02 | 200.95 ± 9.18 | 215.22 ± 3.09 |
| SHR LIN | 193.27 ± 6.32 | 199.50 ± 5.02 | 190.87 ± 10.02 | 192.52 ± 7.21 | 199.34 ± 7.18 |
| SHR LIN\b-CD | 202.11 ± 8.65 | 184.61 ± 7.46* | 170.63 ± 14.46* | 155.66 ± 9.18* | 175.18 ± 7.36* |
| | | | **Heart Rate (HR)** | | |
| Wistar | 319.39 ± 11.58 | - | 369.32 ± 21.00 | - | 344.51 ± 18.81 |
| SHR vehicle | 309.26 ± 13.15 | - | 358.37 ± 15.88 | - | 339.71 ± 15.43 |
| SHR LIN | 342.13 ± 30.71 | - | 362.36 ± 30.59 | - | 368.61 ± 31.65 |
| SHR LIN\b-CD | 401.45 ± 27.48 | - | 294.78 ± 10.88* | - | 299.20 ± 12.87* |

**Supplementary Table S1.** Oral administration effects of LIN e LIN/β-CD at cardiovascular parameters of wistar and SHR.

Note: Values are represented as mean ± standard deviation, respectively (n=6/group). *Statistically different values (α<0,05)

**LIN/β-CD chronic treatment do not affect the body mass of Wistar and SHR**

**Supplementary Figure S4.** Effect of chronic 60-day treatment with LIN/β-CD on body weight gain. Body weight gain of treated Wistar and SHR animals. Groups Wistar (n=5), Vehicle (n=5), SHR LIN 50 mg (n=6) and SHR LIN/β-CD (n=6). Values expressed as mean ± S.E.M.

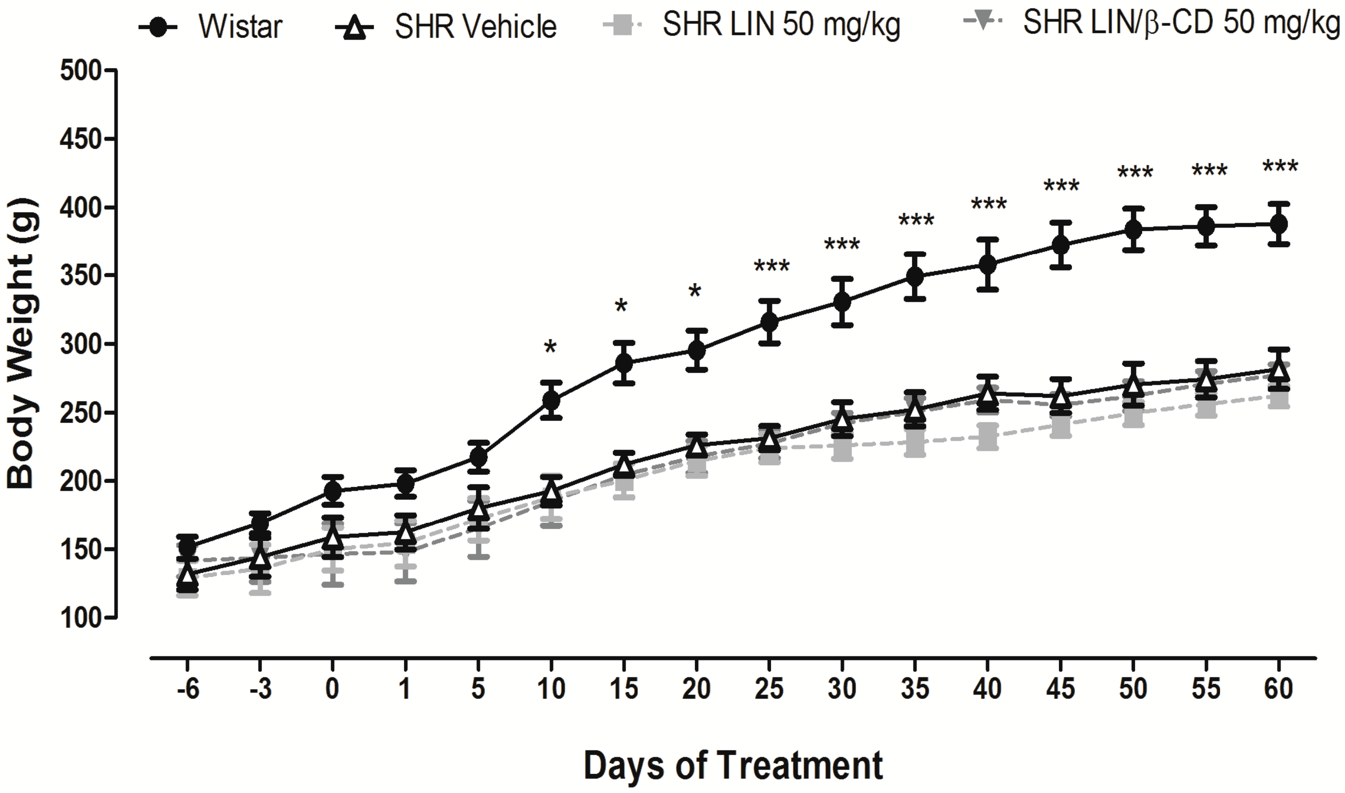


**Final remarks:**

This study advances our understanding of how inclusion complexes can enhance the pharmacokinetic and pharmacodynamic profiles of natural compounds with therapeutic potential. By complexing linalool with β-cyclodextrin, we achieved a significant improvement in systemic bioavailability, cardiovascular efficacy, and safety profile in a hypertensive model. The LIN/β-CD complex promoted sustained antihypertensive activity, improved endothelial function, and mitigated cardiac remodeling without histological evidence of tissue damage. These findings support the utility of β-cyclodextrin as an enabling technology for optimizing the delivery and therapeutic performance of bioactive terpenes. Moreover, this work establishes a solid foundation for the rational design of next-generation oral delivery systems based on natural products, offering new opportunities for safe, effective, and patient-friendly interventions in the treatment of hypertension and related cardiovascular disorders. Ultimately, the translational relevance of this delivery platform encourages further investigation toward clinical development and application in chronic cardiovascular care.
